# NID2 Affects Prognosis of Glioma via Activating the Akt Signaling Pathway

**DOI:** 10.3390/ijms26083859

**Published:** 2025-04-18

**Authors:** Zhangzhang Lan, Yanlin Xiao, Youyou Liao, Xuan Li, Yi Zhang, Huajie Wang, Wenyong Zhang

**Affiliations:** 1 School of Medicine, Southern University of Science and Technology, Shenzhen 518055, China; 11930783@mail.sustech.edu.cn (Z.L.); xiaoyl@mail.sustech.edu.cn (Y.X.); 12133127@mail.sustech.edu.cn (Y.L.); lix33@mail.sustech.edu.cn (X.L.); 12032629@mail.sustech.edu.cn (Y.Z.); 2 Department of Neuroscience, College of Veterinary Medicine and Life Sciences, City University of Hong Kong, Kowloon, Hong Kong SAR, China; huajwang2-c@my.cityu.edu.hk; 3 Key University Laboratory of Metabolism and Health of Guangdong, Southern University of Science and Technology, Shenzhen 518055, China

**Keywords:** NID2, glioma, prognosis

## Abstract

Nidogen-2 (NID2) is a critical component of the extracellular matrix (ECM), which plays a regulatory role in cell adhesion, migration, differentiation, and survival. Previous studies have shown that NID2 is deregulated in several types of cancer, but its role in glioma is unknown. The present study investigated the prognostic value of NID2 in glioma and its associated molecular pathways and functional roles in malignant progression. The performed analyses included investigating the NID2 expression profile using the Cancer Genome Atlas (TCGA), Chinese Glioma Genome Atlas (CGGA), and tumor tissue microarray. The findings demonstrated that NID2 high expression predicts worse patient survival by both univariable and multivariable analyses. There is a strong correlation between NID2 upregulation and tumor grade. In stably NID2-overexpressed glioma cells, RNA-Seq analysis revealed coactivation of oncogenic functional pathways, including cell proliferation, survival, epithelial–mesenchymal transition, ECM organization, and migration. Overexpression of NID2 in U87MG and T98G cells promoted cell proliferation, migration, and invasion. TUNEL assay showed NID2 overexpression protected cells from apoptosis. Western blotting analysis showed activation of Akt and Bcl-xL in NID2-overexpressed cells. Our results show that NID2 is a promising prognostic marker in glioma.

## 1. Introduction

Glioma is the most common primary brain tumor and is difficult to eradicate due to its infiltrative nature [1]. Glioma is graded from 1 to 4 with criteria integrating molecular changes with morphologic features based on the World Health Organization (WHO) classification system [2]. The prognosis of glioma varies greatly based on grade and type [2,3]. Glioblastoma multiforme (GBM) is the grade 4 glioma with the most aggressive behavior, and patients usually die within a year [4]. Grade 2 and 3 glial tumors are considered lower-grade gliomas (LGG), consisting of pleomorphic tumors with variable prognosis [5]. Some LGGs could remain stable for years, while others rapidly progress to GBM within months [6]. For LGG, due to significant variability in clinical behavior and considerable adverse effects associated with the treatment, no consensus on the treatment strategy has been reached [7]. To better manage this devastating disease, there is an urgent need to discover new disease markers to improve prognosis and discover novel treatment targets.

In humans, *NID1* and *NID2* genes encode two nidogen (entactin) proteins, which are key components of the basement membrane and extracellular matrix (ECM) [8,9,10]. Genetic ablation of *NID1* and *NID2* results in limb defects and perinatal lethality [9,11]. Evidence shows that NID1 and NID2 contribute to maintaining stem cell niches [12,13]. In carcinogenesis and cancer progression, it is recognized that the ECM environment in cancer plays a vital role in providing mechanical support and pro-growth, pro-metastatic cell signaling [14]. Both NID1 and NID2 proteins contain integrin binding regions, allowing for engaging with cell surface integrin receptors to affect cell-ECM interaction [15]. Aberrant expression of nidogen proteins has been implicated in several types of cancer, including ovarian [16,17,18], colorectal [19,20], gastric [21], and lung metastasis from breast cancer and melanoma [22]. NID1 was reported to be upregulated in LGG and associated with temozolomide sensitivity [23]. However, the role of NID2 in glioma remains unknown.

This study aimed to investigate the predictive value of NID2 by analyzing the glioma clinical datasets, including GSE16011, GSE7696, GSE4290, the Cancer Genome Atlas (TCGA), the Chinese Glioma Genome Atlas (CGGA), and a glioma tissue microarray (TMA). U87MG and T98G based cell lines stably overexpressed with NID2 were generated to study molecular oncogenic pathways.

## 2. Results

### 2.1. NID2 Upregulation in Glioma

**Figure 1:**
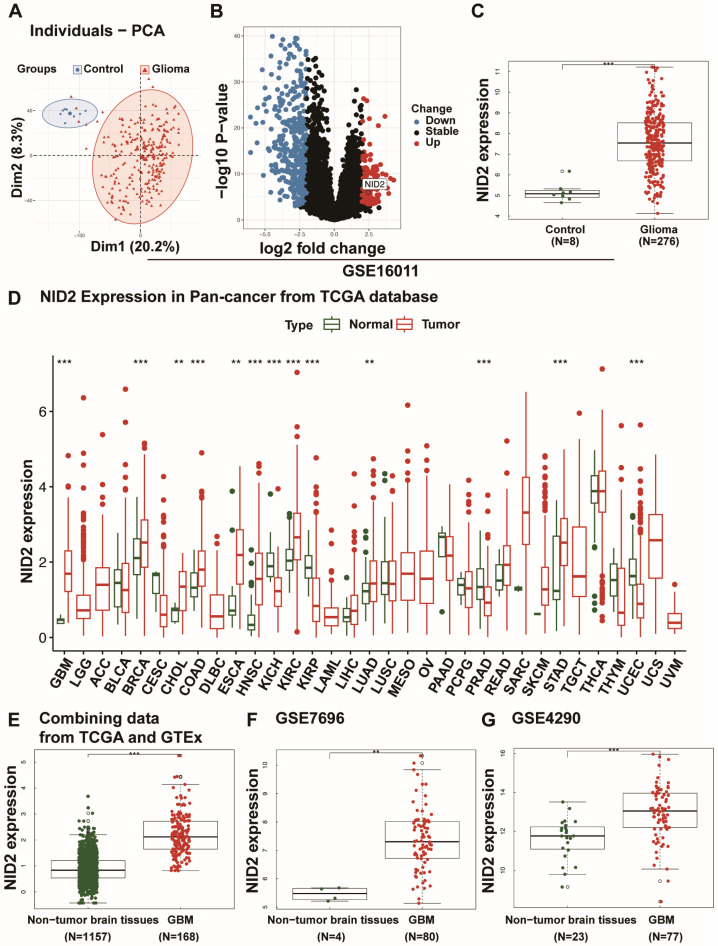
*NID2* was upregulated in various cancer types. (**A**) PCA plot of the 7357 genes filtered by WGCNA in the GSE16011 dataset. (**B**) The volcano plots of 275 DEGs in GSE16011. (**C**) The expression values of *NID2* for gliomas and the controls were compared using the Wilcoxon Rank-Sum test in GSE16011. (**D**) *NID2* expression values in various cancers and adjacent non-cancerous tissue from the TCGA database. The red rectangles represent *NID2* expression in tumor tissues, while the green rectangles represent *NID2* expression in the corresponding non-cancerous tissues. (**E**) *NID2* expression in TCGA GBM samples compared with GTEx brain tissue controls. (**F**,**G**) *NID2* expression in GSE7696 and GSE4290 GBM samples compared with normal controls. The red dots represent *NID2* expression in GBM, and the green dots represent *NID2* expression in the control brain tissues. *** *p* < 0.001, ** *p* < 0.01. Error bars show the standard error. Dim1/2, Dimensionality 1/2.

A weighted gene co-expression network analysis (WGCNA) in the GSE16011 dataset was executed to generate co-expression modules to identify genes related to glioma. Two modules had the highest correlation score and were chosen for further analysis (Supplementary Figure S1) and obtained 7357 genes separated normal from glioma by PCA analysis (Figure 1A). The volcano plots presented the expression of 275 DEGs in glioma, and *NID2* expression was shown to have high expression in glioma compared with the controls (Figure 1B,C). We evaluated the distribution of *NID2* expression levels for pan-cancer in TCGA. *NID2* gene expression is elevated in 9 of 24 cancer types, including GBM, lung adenocarcinoma, colon adenocarcinoma, breast cancer, and renal clear cell carcinoma (*p* < 0.001, Figure 1D). We used GTEx as the normal brain control and showed significant NID2 overexpression in glioma (Figure 1E). Two additional GEO datasets, GSE7696 and GSE4290, were analyzed, and substantial overexpression of *NID2* in GBM was confirmed compared with the non-tumor brain tissue (Figure 1F,G).

### 2.2. NID2 Upregulation Predicts Worse Patient Survival and Higher Tumor Grade

**Figure 2:**
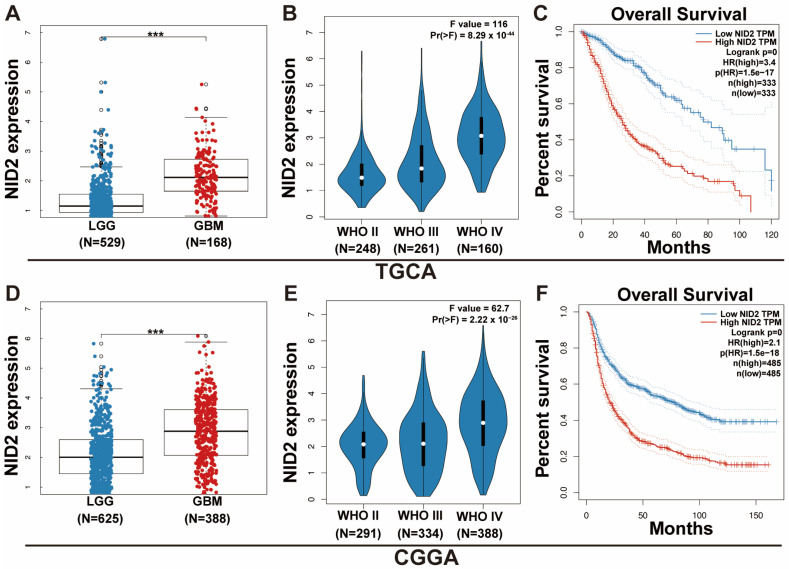
Tumor subtypes and clinical outcomes associated with *NID2* expression in glioma dataset. (**A**,**D**) Wilcoxon rank-sum test was used to analyze the differential expression of *NID2* between GBM and LGG in TCGA (**A**) and CGGA (**D**). (**B**,**E**) Violin plot illustrating *NID2* expression in TCGA (**B**) and CGGA (**E**) dataset according to the grade. (**C**,**F**) Kaplan–Meier survival curves of the TCGA (**C**) and CGGA (**F**) cohort showed that a high level of *NID2* expression was associated with significantly worse overall glioma survival. WHO II, III and IV, World Health Organization grades II, III, and IV. *** *p* < 0.001.

We analyzed the expression levels of *NID2* in the TCGA glioma dataset. The *NID2* expression was significantly higher in GBM than in LGG (*p* < 0.001, Figure 2A). The *NID2* expression level was correlated with higher grade (*p* < 0.001, Figure 2B). A negative correlation was found between *NID2* expression and gene methylation (Supplementary Figure S2). Kaplan–Meier survival curves showed that *NID2* high expression was associated with significantly worse overall survival (OS) for all glioma patients (*p* < 0.0001, Figure 2C). Subgroup analysis showed that *NID2* high expression negatively impacted LGG survival more than GBM (Supplementary Figure S3). Univariate and multivariate Cox regression analyses revealed that *NID2* upregulation is an independent negative factor of survival (HR = 1.23, *p* = 0.002, Supplementary Figure S4)

**Figure 3:**
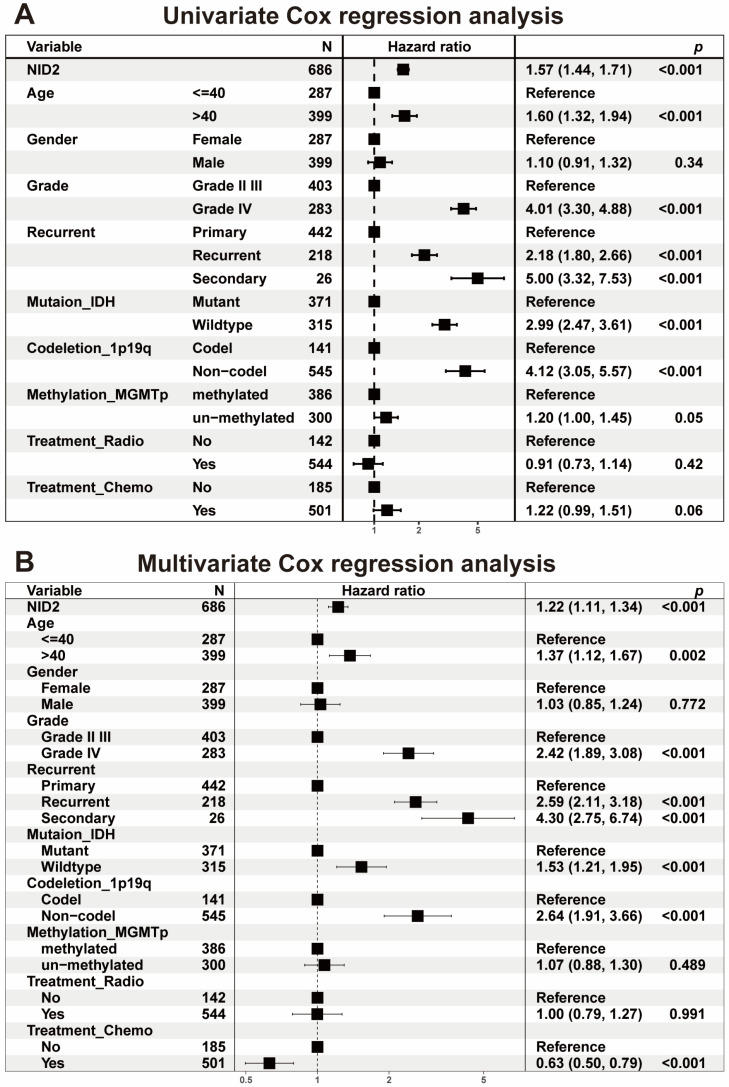
Tumor subtypes and clinical outcomes associated with *NID2* expression in CGGA glioma dataset. (**A**,**B**) Univariate (**A**) and multivariate Cox regression (**B**) analysis demonstrated *NID2* as an independent OS factor in CGGA.

To confirm the findings from TCGA analysis, we analyzed the CGGA glioma dataset, which contains 693 LGGs and 325 GBMs in the Chinese population. Our study showed that *NID2* expression was significantly higher in GBM than in LGG (*p* < 0.001, Figure 2D). The *NID2* expression level was also correlated with a higher grade (*p* < 0.001, Figure 2E) in CGGA. Kaplan–Meier survival analysis showed that high *NID2* expression was associated with significantly worse OS (*p* < 0.0001, Figure 2F). Subgroup analysis showed that *NID2* expression negatively impacted patient survival more in LGG than in GBM (HR 1.27 vs. 1.15, Supplementary Figure S5). High *NID2* expression was associated with worse OS in IDH-wildtype and IDH-mutant LGG subgroups (*p* < 0.0001, Supplementary Figure S6). The univariate and multivariate Cox regression analysis revealed that *NID2* high expression is an independent adverse predictor for patient survival (HR = 1.23, *p* < 0.001, Figure 3).

### 2.3. NID2 Expression Study in Glioma TMA

**Figure 4:**
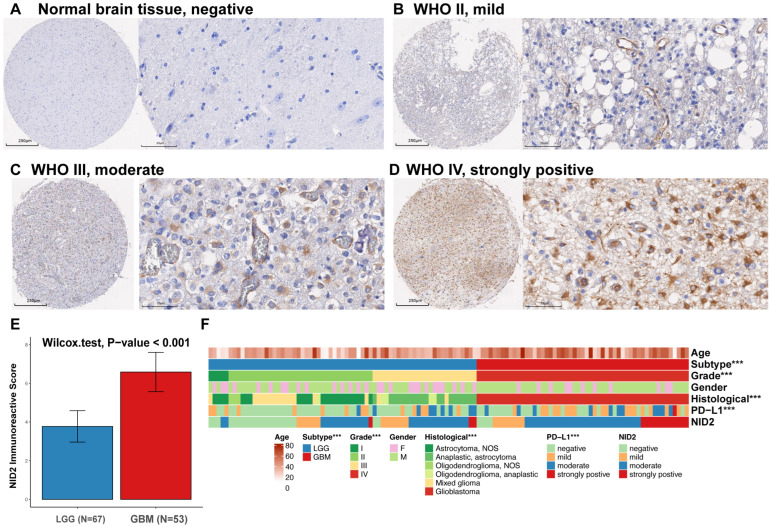
Strong immunoreactivity of NID2 in glioma pathology specimens correlates with high tumor grade. (**A**–**D**). Representative photomicrographs of NID2 IHC staining patterns for normal brain tissue (negative, (**A**)), grade II glioma (mild, (**B**)), grade III glioma (moderate, (**C**)), and GBM (strong, (**D**)) as visualized in 4× (left panel) and 40× (right panel) magnifications. (**E**) Average NID2 immunoreactive score of LGG versus GBM with corresponding 95% confidence interval error bars. The Wilcox test demonstrated a significant difference between the NID2 immunoreactive score in LGGs and GBMs (*p* < 0.001). (**F**) Heatmap of NID2 immunoreactive score distribution according to tumor grade, type, clinical characteristics, and PD-L1 expression in TMA glioma samples. WHO II, III and IV, World Health Organization grades II, III, and IV. *** *p* < 0.001.

To verify gene expression data from TGCA and CGGA datasets, NID2 protein expression was studied by IHC using a glioma TMA. An established IHC quantification method, immunoreactive score (IRS), was used to quantify IHC staining strength based on staining intensity and percentage of positive area. The IRS of each specimen was provided (Supplemental Table S3). A lack of NID2 immunoreactivity was found in the normal brain control samples(Figure 4A). NID2 immunoreactivity increased as glioma grade increased (Figure 4B–D). In grade 4 glioma, there was diffuse cytoplasmic staining of NID2, consistent with its upregulation in glioma cells. In addition, the ECM of grade 4 glioma showed immunoreactivity for NID2, consistent with its function as a secreted protein. Comparing LGG and GBM IRSs showed a statistically significant increase in GMB immunoreactivity compared with LGG (*p* < 0.001, Figure 4E). Clinicopathologic features of TMA samples were presented in the Heatmap format and showed clustering of NID2 upregulation with GBM (Figure 4F).

### 2.4. NID2-Associated DEGs Enrichment Analysis Revealed Key Tumorigenic Pathway Activation

**Figure 5:**
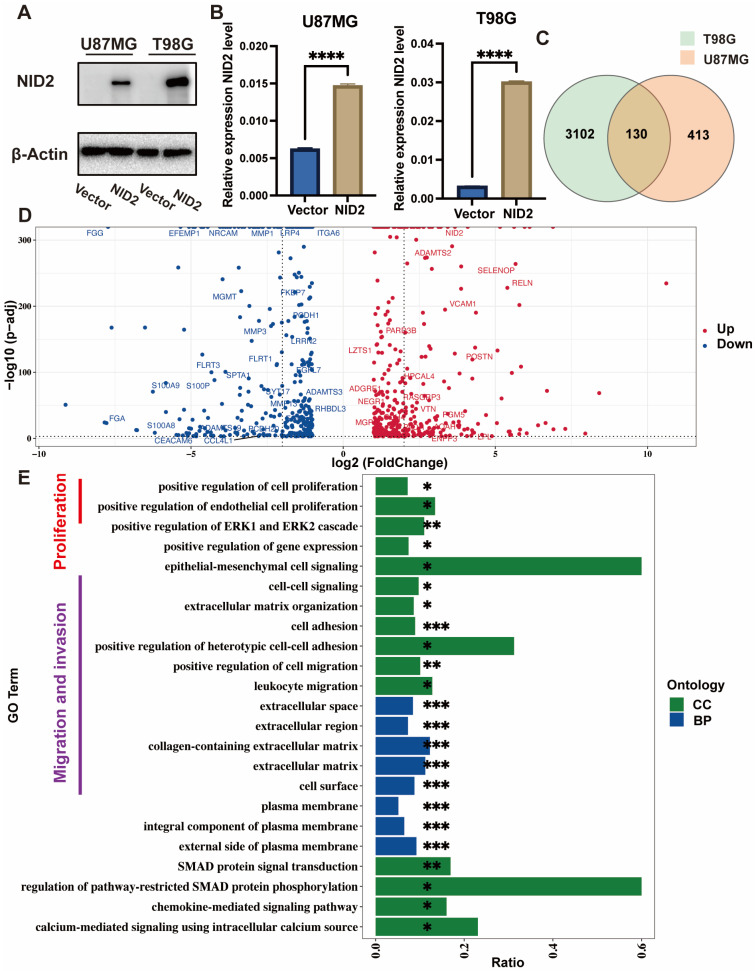
NID2 regulates the proliferation and migration of glioma cells. (**A**) Western blotting results of NID2 expression in the vector controls and NID2-overexpressing U87MG/T98G glioma cells. (**B**) RT-qPCR results of *NID2* expression in the vector controls and NID2-overexpressing U87MG/T98G glioma cells. (**C**) Venn diagram represents genes upregulated in T98G and U87MG glioma cells. (**D**) The volcano plot of DEGs was between the vector controls and the NID2-overexpressing cells. (**E**) Gene ontology enrichment analysis of DEGs regulated by NID2 overexpression. GO enrichment analysis contains biological process (BP) and cellular component (CC). DEGs, differentially expressed genes. **** *p* < 0.0001, *** *p* < 0.001, ** *p* < 0.01, * *p* < 0.05.

To explore molecular pathways activated by the upregulation of NID2 in glioma cells, we created two stable NID2-overexpressing cell lines using U87MG and T98G cells. We confirmed NID2 overexpression by Western blotting and RT-qPCR assays (Figure 5A,B). RNA-Seq was performed to study transcriptomic changes associated with NID2 overexpression in glioma cells. Upregulated genes in the NID2-overexpressing T98G and U87MG cell lines were analyzed and presented in a Venn diagram, which showed co-regulation of 130 genes in the two cell lines (Figure 5C). DEGs were presented in a volcano plot (Figure 5D). Gene ontology enrichment biological process and cellular component pathway analyses of the DEGs revealed that these genes were clustered into positive regulation of proliferation, positive regulation of endothelial cell proliferation, cell survival, epithelial-mesenchymal cell signaling, cell-cell signaling, ECM organization, cell adhesion, and positive regulation of cell migration (Figure 5E), which are essential pathways for malignant proliferation, EMT modulation, angiogenesis, migration, and invasion associated with glioma oncogenesis and progression.

### 2.5. NID2 Overexpression Promoted Proliferation of Glioma Cells

**Figure 6:**
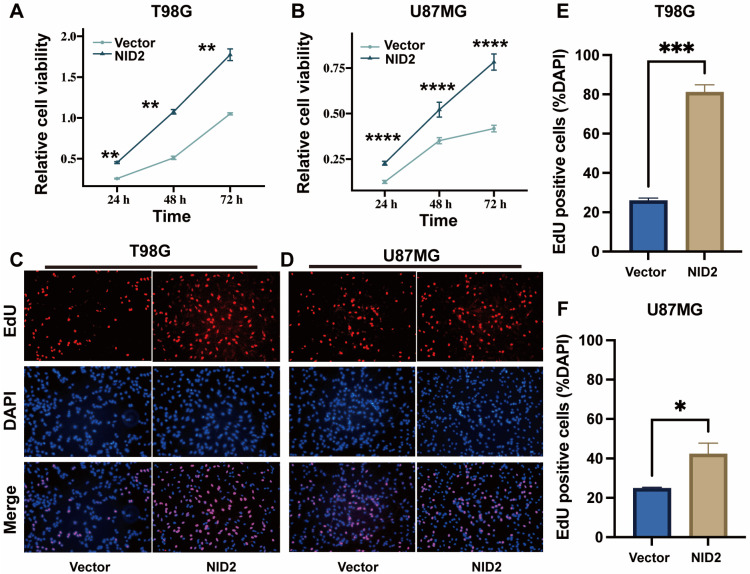
Overexpression of NID2 promoted the proliferation of glioma cells. (**A**,**B**) CCK-8 assay showed that the upregulation of NID2 expression in glioma cells resulted in increased cell proliferation (*n* = 8) in T98G (**A**) and U87MG (**B**) glioma cells. (**C**,**D**) The represented image of the EdU assay showed that NID2 overexpression in T98G and U87MG cells promoted cell proliferation (Magnification: 10×, *n* = 3). (**E**,**F**) Histograms represent the percentage of the EdU-positive cells. **** *p* < 0.0001, *** *p* < 0.001, ** *p* < 0.01, * *p* < 0.05.

The CCK8 assay results showed that cell proliferation was significantly enhanced in the NID2-overexpressing T98G and U87MG cells compared with the vector controls (Figure 6A,B). The EdU single cell proliferation assay confirmed the result (Figure 6C–F).

### 2.6. NID2 Overexpression Promoted Migration and Invasion of Glioma Cells

**Figure 7:**
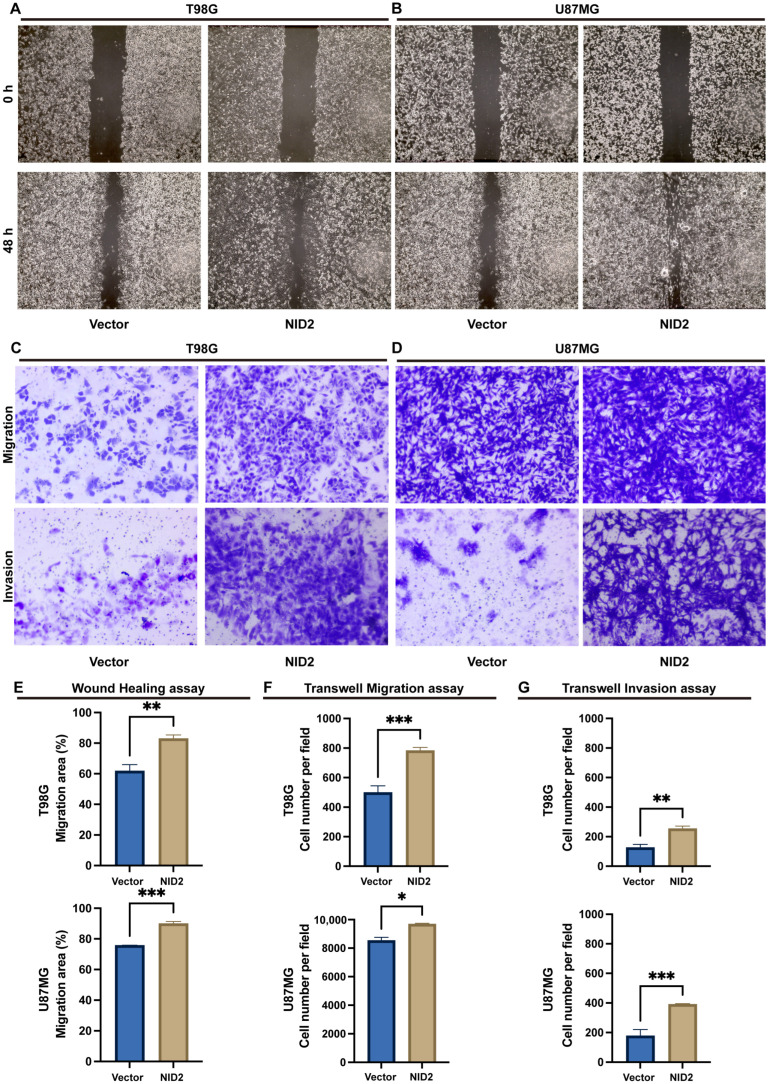
Overexpression of NID2 promoted migration and invasion of glioma cells. (**A**,**B**) Wound healing assay showed overexpression of NID2 enhanced glioma cell migration (representative images of wound scratch). (**C**,**D**) Overexpression of NID2 promoted the migration and invasion of glioma cells examined by transwell assay. (**E**–**G**) Histograms represent the analysis of the wound healing rate (**E**), migration cell number (**F**), and invasion cell number (**G**). *** *p* < 0.001, ** *p* < 0.01, * *p* < 0.05.

The scratch wound healing assay showed that overexpression of NID2 in T98G and U87MG cells promoted cell migration compared to the control group (Figure 7A,B). Moreover, the transwell migration assay also revealed that overexpression of NID2 in T98G and U87MG cells increased the number of tumor cells migrated through the membrane (Figure 7C,D, upper). A transwell invasion assay was performed to test whether NID2 overexpression influences the invasive ability of glioma cells. The number of tumor cells invaded through the Matrigel-coated transwell inserts was significantly increased by NID2 overexpression in T98G and U87MG cells (Figure 7C,D, lower).

### 2.7. Akt Was Activated in NID2-Overexpressing Cells

**Figure 8:**
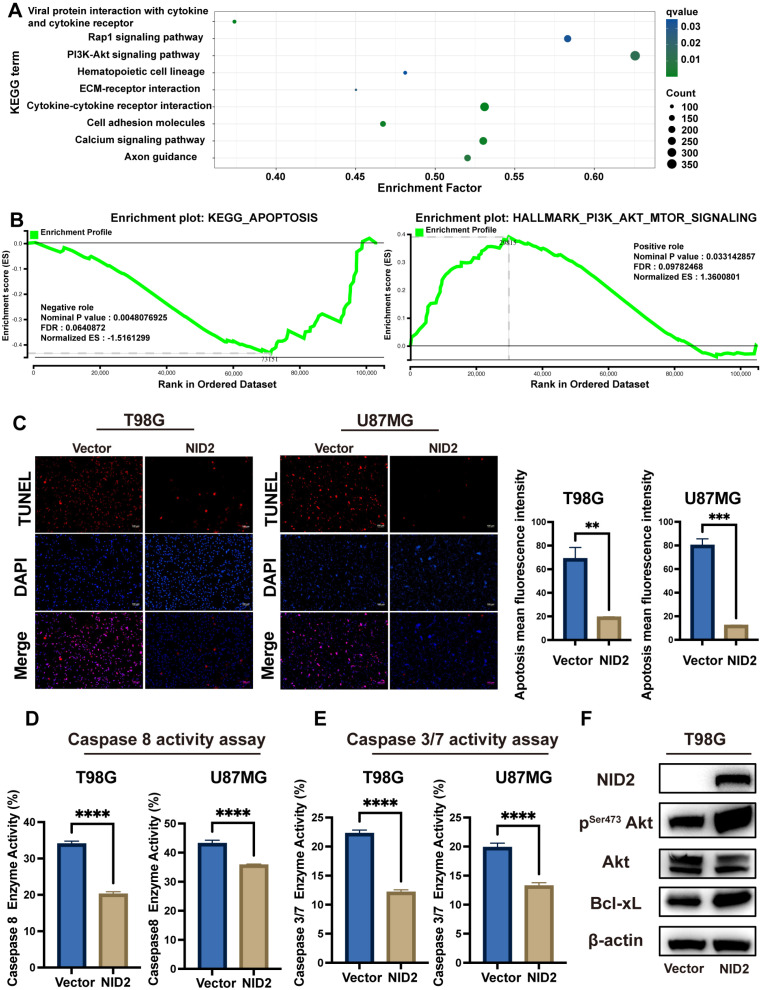
Overexpression of NID2 protected against glioma cell apoptosis. (**A**) KEGG enrichment analysis of the DEGs between the vector control groups and NID2-overexpressing T98G/U87MG glioma cells revealed significant activation of Akt signaling and extracellular matrix remodeling pathways. (**B**) GSEA demonstrated that NID2-overexpressing glioma cells exhibited enhanced negative regulation of apoptosis and Akt pathway activation. (**C**) The TUNEL assay revealed a significant decrease in apoptotic cells in the NID2-overexpressing cells compared to the vector controls (Scale bar: 20 µm). (**D**,**E**) Caspase 8 and caspase 3/7 activity assays showed the apoptosis proteins were downregulated in the NID2 overexpression group (*n* = 8). (**F**) Western blotting of apoptosis markers in T98G glioma cells. **** *p* < 0. 0001, *** *p* < 0.001, ** *p* < 0.01.

KEGG pathways enrichment analysis of RNA-Seq data of the NID2-overexpressing T98G and U87MG cells showed that the most enriched pathways were PI3K-Akt, Rap1, cytokine-cytokine receptor interaction, cell adhesion, calcium signaling, and axonal guidance. (Figure 8A). Furthermore, GSEA analysis suggested that processes such as apoptosis genes were negatively regulated with NID2 overexpression and PI3K-Akt signaling pathway genes were activated by NID2 overexpression in glioma cells (Figure 8B).

We followed up the KEGG and GSEA analysis with assays to evaluate cellular apoptosis. The TUNEL assay revealed that apoptosis was significantly reduced in the NID2-overexpressing cells (Figure 8C). The apoptotic proteins activity assays (caspase 8 and caspase 3/7) revealed that the activity of apoptosis proteins was downmodulated in the NID2-overexpressing cells (Figure 8D,E). Furthermore, Western blotting analysis showed that the expression of p-Akt and anti-apoptotic protein Bcl-xL were upregulated in the NID2-overexpressing cells compared with the vector control groups (Figure 8F).

### 2.8. Akt Inhibitor 124005 Reversed Anti-Apoptotic Effect of NID2 Overexpression

**Figure 9:**
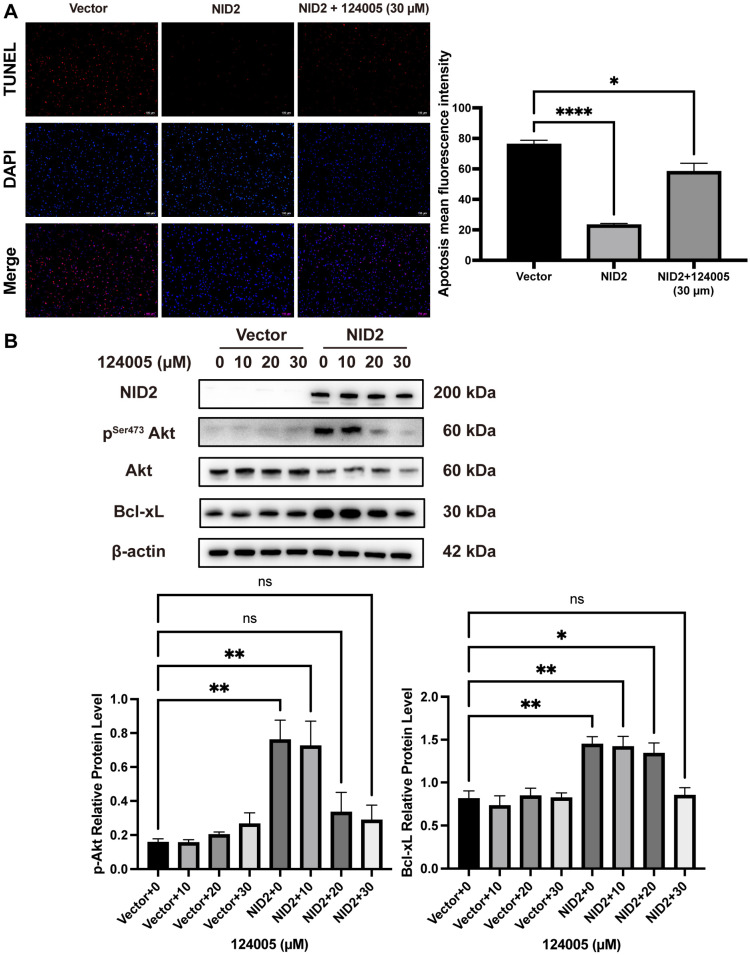
Blockage of Akt signaling dampened the anti-apoptic effect of NID2 overexpression. (**A**) Comparison of apoptotic cells visualized by the TUNEL assay. (**B**) The activation of Bcl-xL anti-apoptic protein by NID2 overexpression could be reversed by Akt inhibition. **** *p* < 0.0001, ** *p* < 0.01, * *p* < 0.05.

We used a specific signal transduction inhibitor to test the pivotal role of Akt phosphorylation affecting anti-apoptotic signaling in the NID2-overexpressing cells. Akt1 inhibitor 124005 was added in the experimental group and then TUNEL assay and Western blotting were performed. Cell lysates were harvested 18 h after being treated with 10, 20, and 30 µM of 124005 for the analysis of p-Akt, Akt, and Bcl-xL expression. As shown in Figure 9A, the anti-apoptotic effect caused by overexpression of NID2 can be largely reversed by the addition of 30 µM of 124005. As shown in Figure 9B, overexpression of NID2 significantly elevated the protein expression of p-Akt and anti-apoptotic protein Bcl-xL. Meanwhile, treating with 20 µM and 30 µM of 124005 could decrease the expression of p-Akt and Bcl-xL in the NID2-overexpressing cells as compared with the vector controls.

## 3. Discussion

Glioma is a heterogenous group of entities with many histology subtypes and a complex molecular mutation landscape, which hinders more precise prediction of tumor behavior and patient survival [7,24,25,26]. The tumor microenvironment is critical in cancer progression and is a potential target for cancer therapy [27]. NID1 and NID2 proteins are functional components of ECM. In addition to their roles in supporting the basement membrane [13], they also play a role in cell–cell signaling, such as regulating cellular differentiation, proliferation, neo-vascularization, migration, and tissue regeneration, by binding to cell surface integrin receptors [28]. It has been recognized that dysregulation of ECM composition, structure, stiffness, and abundance modulates the hallmarks of cancer and contributes to neoplastic progression [28,29]. Secreted NID1 was shown to act as a paracrine to induce EMT and promote colorectal cancer progression [20]. Yu et al. reported that NID2 overexpression was found in gastric cancer tissue, and gene pathway analysis revealed that NID2 overexpression may be involved in protein digestion and absorption, cellular amoebiasis, PI3K-Akt-signaling pathway, focal adhesion, and ECM-receptor interaction [21]. Several groups have provided evidence that serum NID2 levels could serve as a potential ovarian cancer screening marker and higher serum levels of NID2 were correlated with higher disease stages [16,17].

Our current study is the first to show that *NID2* overexpression in glioma is correlated with higher tumor grade and poor patient survival. Using the TCGA database, *NID2* gene overexpression was shown to be a significant adverse survival factor by univariate and multivariate Cox analysis (*p* < 0.01, Supplementary Figure S4). This result was confirmed using the CGGA database (*p* < 0.001, Figure 3). Using both CGGA and TCGA glioma databases which together contain 1710 glioma cases allowed us to draw conclusions across diverse ethnic backgrounds. A TMA with 120 glioma samples was analyzed and showed high NID2 protein expression levels were correlated with GBM status (Figure 4). In GBM, NID2 showed a diffuse, strong cytoplasmic staining pattern, consistent with upregulation. NID2 immunoreactivity was seen surrounding the vessel structures in the LGGs, supporting its structural role as component of the basement membrane. Our immunohistochemistry study of NID2 in TMA confirmed the bioinformatic findings and pointed to the possibility of using NID2 IHC in the identification of higher-grade glioma in the practice of pathology diagnosis.

LGG, which includes both WHO grade II and III tumors, presents a diagnostic and treatment challenge due to its diverse morphologic appearance and clinical outcomes. Although, in the recent years, molecular studies, such IDH mutation and 1p/19q codeletion status, help to stratify patients’ risk profiles, there is still great need to provide better prognostic markers based on molecular studies. Here, we demonstrated that *NID2* overexpression was an additional independent adverse prognostic factor for all gliomas, and it had an additive negative prognostic effect in addition to IDH mutation and 1p/19q codeletion status analyses for patient survival (Figure 3). Thus, we can further improve glioma patient prognosis by adding NID2 expression to the current known list of molecular prognostic markers (Supplementary Figure S6).

To understand the molecular pathways of NID2 overexpression in glioma progression, we created two stable cell lines that overexpress NID2. RNA-Seq followed by GO, KEGG, and GSEA analyses showed that NID2 overexpression in glioma cells is accompanied by activation of pathways involved in promoting cell proliferation, endothelial cell proliferation, cell survival, epithelial–mesenchymal cell signaling, cell–cell signaling, ECM organization, cell adhesion, and cell migration (Figure 5E and Figure 8A). Our experiments using NID2-overexpressing glioma cell lines confirmed that NID2 overexpression leads to more robust proliferation, migration, invasion, and anti-apoptosis activities. By KEGG and GSEA analyses, the most strongly activated signaling pathway in the NID2-overexpressing glioma cells is the PI3K/AKT signaling pathway, which lies at the heart of carcinogenesis and cancer progression, including promoting proliferation, resisting apoptosis, promoting EMT, angiogenesis and maintaining cancer stemness [30,31,32,33]. Results from in vitro assays supported the gene pathway activation data that NID2 overexpression promoted glioma cell proliferation, migration, and invasion (Figure 6 and Figure 7). The activation results of the GSEA pathways were confirmed by Western blotting analysis, where p-Akt and Bcl-xL were upregulated in the NID2-overexpressing glioma cells (Figure 8). The Akt inhibitor 124005 blocks Akt signaling pathway by inhibiting the generation of PIP3 by PI3K [34]. When we added the Akt inhibitor 124005, the apoptosis level of the NID2-overexpressing cells returned to the same level as that of the vector control cells (Figure 9). These results suggest that NID2 overexpression activates PI3K/AKT signaling pathway, leading to malignant transformation and progression.

Our current study shows that high expression of NID2 in glioma is an adverse prognostic factor. Since LGGs cover a large spectrum of gliomas and there is uncertainty regarding why some LGGs progress to GBM rapidly, and others do not, further subclassification of LGGs based on novel molecular marker studies is important. Our study suggests that the characterization of an immunoreactivity score using NID2 IHC on pathology specimens provides a potential method for assessing glioma aggressiveness. Further confirmatory studies using additional clinical samples from different study groups will be the necessary next step in the investigation of its clinical value. Several studies have shown promising results that high serum NID2 level could serve as a predictive marker for higher-stage ovarian cancer, particularly the serous type [35]. It is of great interest to conduct studies assessing serum and cerebrospinal fluid (CSF) NID2 levels as predictive cancer markers during the clinical workup of glioma patients.

## 4. Materials and Methods

### 4.1. Cell Lines, Cell Culture, and Transfection

Human 293T and glioblastoma cell lines U87MG and T98G were purchased from the American Type Culture Collection (Manassas, VA, USA). Cells were cultured in Dulbecco’s modified Eagle’s medium (Gibco, Thermo Fisher Scientific, Waltham, MA, USA) with 10% fetal bovine serum (Gibco, Thermo Fisher Scientific) and 1% penicillin and streptomycin (Gibco, Thermo Fisher Scientific) maintained in 5% CO_2_ incubator at 37 °C.

U87MG and T98G cells with stable NID2 overexpression were achieved using the lentiviral transduction method. *NID2* or vector plasmids were generated by subcloning the PCR-amplified human full-length cDNA encoding *NID2* (NM_007361) or the empty vector into a lentiviral vector (pCDH-3xFLAG-GFP-puroR, Addgene, Watertown, MA, USA). 293T cells were transfected with 1 µg packaging plasmid (psPAX2, Addgene), 500 ng envelope plasmid (pMD2.G, Addgene), and 1 µg transfer plasmids (NID2/vector) in a 6 cm dish with 60% confluence using lipofectamine 3000 transfection reagent (Thermo Fisher Scientific). After 48 h, viruses were collected, filtered, concentrated, and used to infect U87MG and T98G cells. The infected cells were selected with puromycin (1 µg/mL) for one month. The cells were designated as U87MG-NID2, U87MG-vector, T98G-NID2, T98G-vector, respectively.

### 4.2. Datasets and Bioinformatic Processing

A total of 2263 glioma sample data from five independent datasets were used in our study. TCGA database contains 529 LGGs and 168 GBMs (UCSC Xena https://xenabrowser.net/, accessed on 16 December 2023) [6,36]. The gene expression profiles (RPKM: reads per million reads per kilobase), methylation profiles, and clinical information were integrated through ID numbers. The RPKM of log2 was transformed to TPM and then normalized with the scale method through the “limma” package [37] with R software 4.2.1. The DNA methylation and mRNA expression profiles, survival outcomes, and clinical characteristics of 693 LGGs and 325 GBMs were downloaded from the CGGA website (http://www.cgga.org.cn/, accessed on 14 November 2023) [38]. Gene expression profiles from Gene Expression Omnibus (GEO) datasets reposited under accession numbers GSE16011, GSE7696, and GSE4290 (https://www.ncbi.nlm.nih.gov/geo/, accessed on 14 November 2023) [39,40,41] were obtained through the “GEOquery” package in R [42]. We first conducted weighted gene co-expression network analysis (WGCNA) [43]. The “limma” package was used to screen out differential expression genes (DEGs, |logFC| > 2, *p* < 0.001) in glioma samples compared with the control samples in GSE16011, which contains 276 glioma samples and eight control samples. GSE7696 contains 80 GBM samples and four normal brain tissues. GSE4290 contains 77 GBM samples and 23 normal samples. Normal brain tissue expression profile was downloaded from Genotype-Tissue Expression (GTEx, https://gtexportal.org/home/, accessed on 14 November 2024), which contains 1157 normal brain tissue mRNA expression data [44]. We performed NID2 differential-expression analysis with TCGA and GTEx RNA-Seq data between glioma and normal tissue brain. The clinicopathological characteristics of the patients used in this study were summarized in Supplementary Tables S1 and S2.

### 4.3. Glioma Tissue Microarray

A tissue microarray containing 120 glioma samples (67 LGGs and 53 GBMs) and three normal brain tissues were obtained from Shanghai Outdo Biotech. All procedures involving human participants in this study followed the Declaration of Helsinki (as revised in 2013). The Ethics Committee approved this study (No. YB M-05-02). Individual consent for this retrospective analysis was waived. The clinicopathological characteristics of these patients are summarized in Supplementary Table S1.

### 4.4. Survival Analysis

Survival analysis (log-rank test and Kaplan–Meier) was used to compare the survival outcomes between the two groups. The univariate and multivariate Cox regression models were performed to evaluate the impact on survival. A *p*-value of 0.05 or less is considered statistically significant.

### 4.5. Western Blotting

Cells were harvested, lysed in RIPA buffer (Solarbio Life Science, Beijing, China) containing protease inhibitors, and quantified using a BCA Protein Assay Kit (Thermo Fisher Scientific). The cells were lysed and loaded onto a 4–12% SurePAGETM Gel (GenScript, Piscataway, NJ, USA). The PVDF membrane (Merck, Millipore, Burlington, MA, USA) was probed with anti-NID2 (Abcam, Cambridge, UK, ab232883, rabbit 1:1000), anti-β-actin (Proteintech, Wuhan, China, 66009-1, mouse 1:10,000), anti-N-cadherin (Proteintech, 66219-1-Ig, mouse 1:1000), anti-Akt/p-Akt (S473) (Cell Signaling Technology, Danvers, MA, USA, #4691, #4060, rabbit 1:1000), anti-vimentin (Abcam, ab92547, rabbit 1:1000), and anti-Bcl-xL (Cell Signaling Technology, #2764, rabbit 1:1000). Protein expression was detected using HRP-linked anti-rabbit IgG (Cell Signaling Technology, CST, #7074, 1:5000) or HRP-linked anti-mouse IgG (Cell Signaling Technology, CST, #7076, 1:5000) antibodies. The immune-reactive bands were detected using ECL (Merck, Millipore) and visualized using the ChampChemi Chemiluminescence instrument (SageCreation, Beijing, China).

### 4.6. Reverse Transcription and RT-qPCR

Total RNA was extracted from cells using MolPure^®^ Cell/Tissue Total RNA Kit (Yeasen, Shanghai, China, 19221ES50) and reverse transcribed with Transcriptor First Strand cDNA Synthesis Kit (Roche, Basel, Switerland, 04897030001) following the manufacturer’s instructions. RT-qPCR was performed using the SYBR Green PCR Master mix with LightCycler 96 (Roche). The primer sequences for *NID2* were forward—GGGAGATGGACGGAACTGTG and reverse—CACTCCGGCACTCACACCTG. The primer sequences for GAPDH were forward—AATATCATCCCTGCTTCTACTGG and reverse—CATACTTGGCAGGTTTCTCCA.

### 4.7. Cell Counting Kit-8 and EdU Assays

For the Cell Counting Kit-8 (CCK-8) assay, cells were seeded in 96-well plates and cultured at 37 °C with 5% CO_2_. Ten percent CCK-8 reagent (Yeasen, Shanghai, China, 40203ES88) was added to each well and incubated for one hour according to the manufacturer’s protocol after culturing cells for 0 h, 24 h, 48 h, and 72 h, respectively. The mean absorbance at 450 nm was measured with the microplate reader (BioTek, Winooski, VT, USA). EdU assay was performed to detect the cell proliferation. Cells were seeded in 24-well plates and cultured at 37 °C with 5% CO_2_ after 48 h. Cells were incubated with EdU for two hours before fixation and permeabilization, and EdU staining was performed with a BeyoClickTM EdU-555 kit (Beyotime, Shanghai, China, C0075S). The cell nuclei were stained with DAPI (Cell Signaling Technology, #4083, 1:10,000) for 5 min. The proportion of cells that incorporated EdU was determined through fluorescence microscopy. ImageJ (version: 1.54f) was used to count the total number of cells and the number of proliferating cells. Proliferation was analyzed using the mean number of cells in three fields for each sample. A total number of blue fluorescent-labeled cells and proliferating red fluorescent-labeled cells, cell proliferation rate (%) = a number of red fluorescent cells/number of blue fluorescent cells × 100%.

### 4.8. Wound Healing and Transwell Assays

In wound healing assay, cells were seeded in 24-well plates with five lines on the back. When cells reached 80~90% confluence, artificial gaps were scratched using a 200 µL pipette tip, then washed three times with PBS and incubated with serum-free DMEM medium. After 24 h, migration according to the position of the line was assessed microscopically. For Transwell assay, cells (2.0 × 10^4^ cells) were seeded in serum-free DMEM into the upper part of the Cell Culture Insert (Corning, NY, USA, 354262) with or without Matrigel (Corning, 354262). The lower chamber contained 800 μL of DMEM medium supplemented with 20% FBS. After 24 h incubation for migration and 48 h for invasion, cells in the upper chamber were carefully wiped off. Cells on the lower side were fixed with 4% paraformaldehyde (Solarbio Life Science) and stained with crystal violet. Three random fields were selected and measured using microscopy.

### 4.9. Apoptosis Assay

Cell apoptosis was evaluated using the caspase 3/7, caspase 8 activity assay, and TUNEL assay. Caspase 3/7 activity assay was carried out using the Caspase-Glo^®^ 3/7 Assay Kit (Promega Corporation, Madison, WI, USA, G7791). Caspase 8 activity assay used the Caspase-Glo^®^ 8 Assay (Promega, G8202). One Step TUNEL Apoptosis Assay Kit (Beyotime, C1086) is used to detect cell apoptosis in cell slides. Akt inhibitor (Calbiochem 124005) was obtained from Calbiochem (San Diego, CA, USA).

### 4.10. Transcriptome Sequencing (RNA-Seq) Analysis

The total RNAs from the NID2-overexpressing T98G and U87MG cells and corresponding vector-only cells were isolated and underwent quality control. BGI-Wuhan performed the whole transcriptome libraries and deep sequencing. The genes with fold change > 2, adjusted *p*-value < 0.05, were identified as differentially expressed genes (DEGs). GO and GSEA was performed for functional annotation and enrichment analysis to assess the functional features of differentially expressed genes. The functional terms with adjusted *p*-value < 0.05 were identified as correlated terms.

### 4.11. Immunohistochemistry

Immunohistochemical staining was performed using standard procedures. NID2 was detected using an anti-NID2 antibody (Santa Cruz Biotechnology, Dallas, TX, USA, sc-373859) at 1:500 dilution according to the manufacturer’s instructions. Briefly, the processed sections were blocked with 5% BSA used for section blocking, and an anti-NID2 antibody was used as the primary antibody incubated overnight at 4 °C. Next. The DAB Detection Kit was used to develop a staining signal, counterstained with hematoxylin, and captured with Leica Aperio VERSA. Normal brain tissue served as the negative control. For tumor tissues, the staining intensity was estimated as negative, weak, moderate, and strong, corresponding to the scores of 0, 1, 2, and 3. The percentage of positive cells was calculated following the criteria: 4, >80%; 3, 51~80%; 2, 10~50%; 1, <10%; 0, 0%. Three independent pathologists blinded to the identity of the sample evaluated the percentage of positive cells and staining intensity. The immunoreactive score (IRS) was calculated by multiplying the staining intensity score (0~3) by the positive cell proportion score (0~4) [45]. Immunoreactivity was determined by using the IRS with the following criteria: negative (0~1), mild (2~3), moderate (4~8), and strongly positive (9~12) [45].

### 4.12. Immunofluorescence

Cells were fixed with 4% paraformaldehyde (Solarbio Life Science) for 15 min and then washed with PBS. Cells were treated with 10% BSA for 30 min at room temperature, followed by permeabilization. Cells were incubated overnight with the anti-vimentin (Abcam, ab92547, rabbit 1:200) as primary antibody at 4 °C. A secondary goat anti-rabbit antibody conjugated with Alexa Fluor 594 (Abcam, ab150080, 1:1000) was applied. Images were taken with a confocal laser scanning microscope (LSM780, Carl Zeiss AG, Oberkochen, Germany).

### 4.13. Statistics

Statistical analyses were conducted using R software (version R 4.1.2). The Student’s *t*-test was applied to compare continuous variables between the two groups. Wilcoxon rank-sum test and one-way ANOVA were used to calculate the relationship between NID2 expression and pathological characteristics. Pearson’s correlation analysis was adopted to determine the correlation between two variables. Each experiment was independently performed at least three times with similar results. All tests were two-sided. Statistical significance was set at *p* < 0.05.

## Data Availability

Publicly available datasets were analyzed in this study; these can be found in The Cancer Genome Atlas (https://xena.ucsc.edu/) (TCGA-LGG and GBM), Chinese Glioma Genome Atlas (http://www.cgga.org.cn/) (CGGA) and the NCBI Gene Expression Omnibus. All the analysis processes have been uploaded to the GitHub website (https://github.com/zhangzhanglan/Glioma_predict_outcomes) (accessed on 14 April 2025).

## Supplementary Materials

The following supporting information can be downloaded at: https://www.mdpi.com/article/10.3390/ijms26083859/s1.

## Abbreviations

The following abbreviations are used in this manuscript:

NID2	Nidogen-2
ECM	Extracellular matrix
TCGA	Cancer Genome Atlas
CGGA	Chinese Glioma Genome Atlas
GBM	Glioblastoma multiforme
LGG	Lower-grade gliomas
WGCNA	Weighted gene co-expression network analysis
TMA	Tissue microarray
