# Supplementary material for: NID2 Affects Prognosis of Glioma via Activating the Akt Signaling Pathway
Source: Int J Mol Sci. 2025 Apr 18;26(8):3859. doi: 10.3390/ijms26083859 (PMC12028320; doi:10.3390/ijms26083859)
Supplement: Supplementary file 1 [file ijms-26-03859-s001.zip › ijms-3530834-supplementary.pdf]

Supplementary Material

1 Supplementary Figures and Tables

1.1 Supplementary Figures

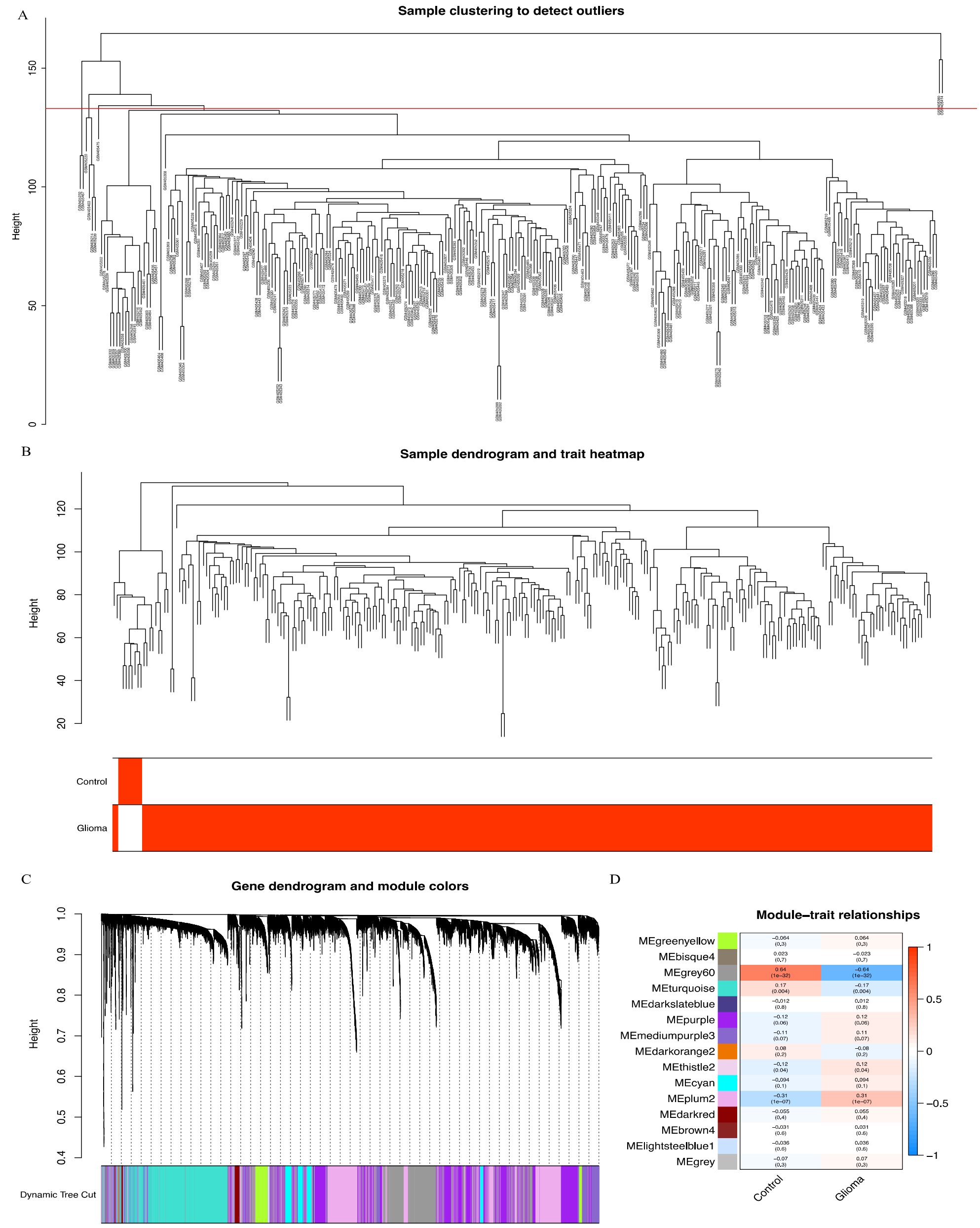

Supplementary Figure S1. WGCNA was constructed and screened gliomas-related genes in the GSE16011 dataset. (A) Samples clustering to identify outliers. (B) Samples clustering and groups. (C) A total of 15 co-expressed modules were identified by setting MEDissThres as 0.6 and minModuleSize as 25. (D) Module-trait heatmap showed the plum2 and grey60 modules correlation with gliomas samples.

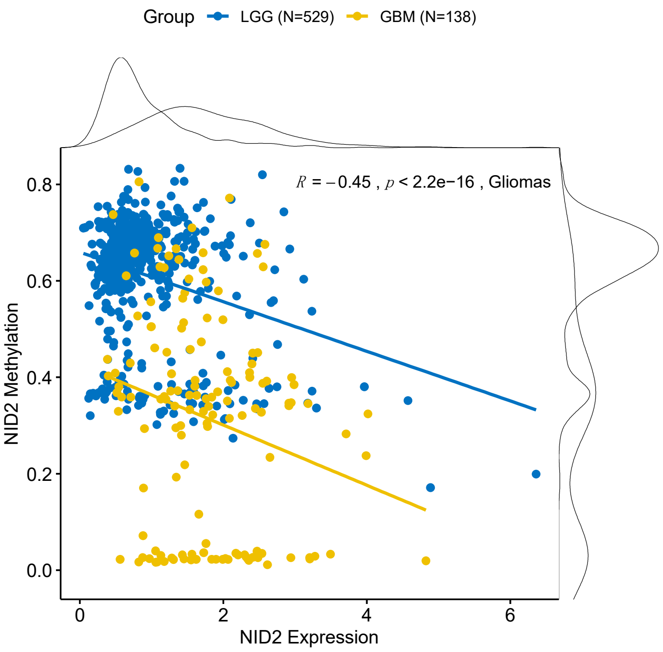

Supplementary Figure S2. NID2 methylation levels inversely correlated with gene expression in both GBM and LGG groups.

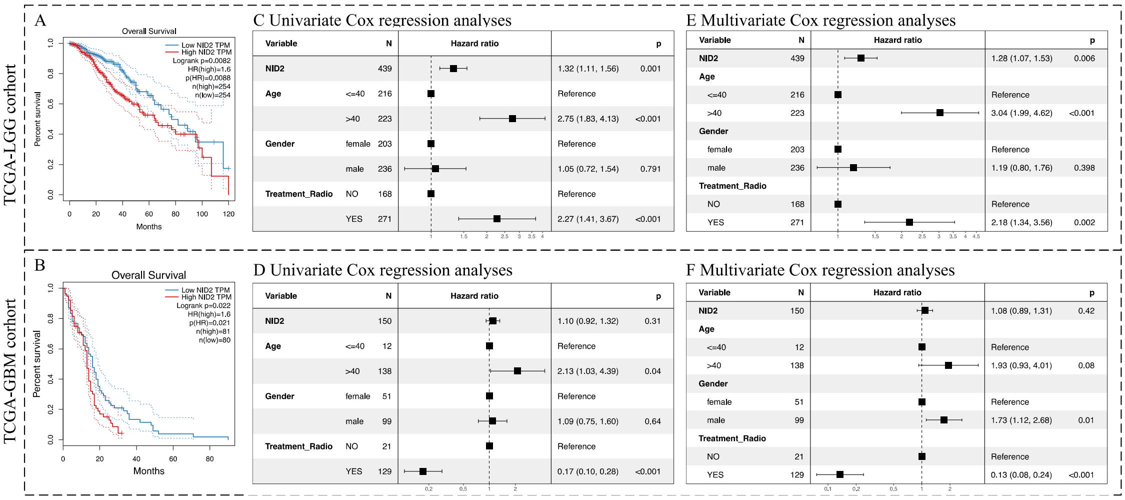

Supplementary Figure S3. Predictive value of NID2 overexpression in TCGA for LGG and GBM subgroups. (A-B) Kaplan-Meier survival curves of the LGG and GBM subgroups in TCGA based on NID2 expression. (C-D) Univariate cox regression analysis in LGG and GBM subgroups. (E-F) Multivariate cox regression analysis in LGG and GBM subgroups.

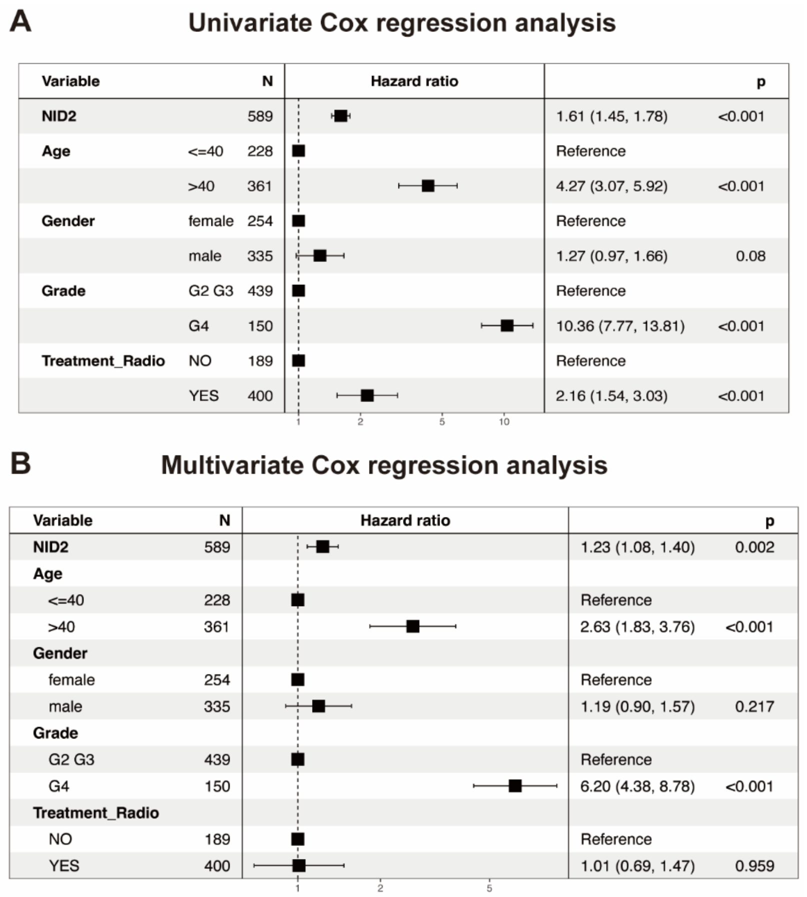

Supplementary Figure S4. Tumor subtypes and clinical outcomes associated with NID2 expression in TCGA glioma dataset. (A, B) Univariate (A) and multivariate Cox regression (B) analysis demonstrated NID2 as an independent OS factor in TCGA.

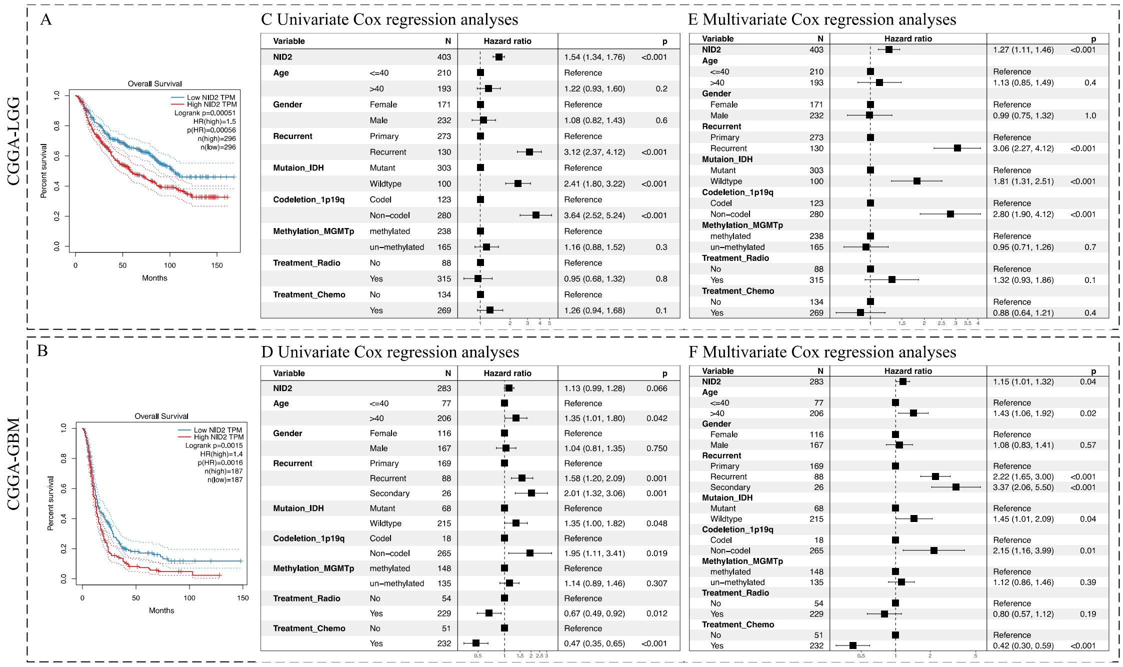

Supplementary Figure S5. Predictive value of NID2 overexpression in CGGA for LGG and GBM subgroups. (A-B) Kaplan-Meier survival curves of the LGG and GBM subgroups in CGGA based on NID2 expression. (C-D) Univariate cox regression analysis in LGG and GBM subgroups. (E-F) Multivariate and cox regression analysis in LGG and GBM subgroups.

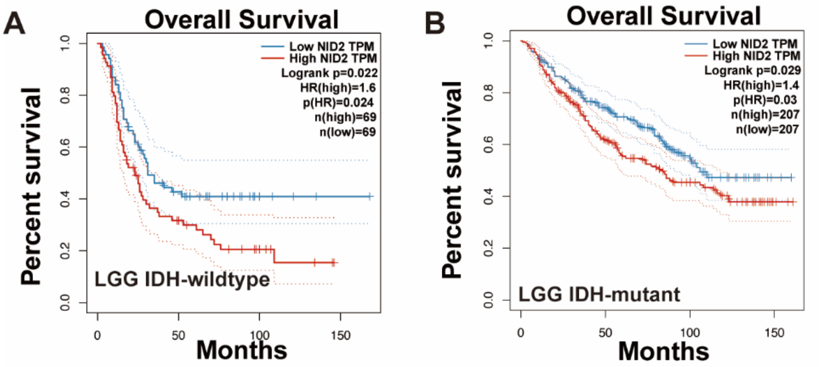

Supplementary Figure S6. Kaplan-Meier survival curves of NID2 overexpression in CGGA based on IDH status. (A) Kaplan-Meier survival analysis showed significantly worse OS in the NID2 high expression group compared with the low NID2 expression group in the CGGA LGG IDH-wildtype subgroup. (B) Kaplan-Meier survival analysis showed significantly worse OS in the NID2 high expression group compared with the low NID2 expression group in the CGGA LGG IDH-mutant subgroup.

1.2 Supplementary Tables

Supplementary Table S1. Clinicopathologic characteristics of TCGA, CGGA, and TMA datasets.

**Datasets**	**TCGA**	**CGGA**	**TMA**
Total No. of patients	1122	1018	120
**Patient demographics**			
Gender, No. (%)			
Female	460 (41)	417 (41)	39 (33)
Male	651 (58)	601 (59)	81 (68)
No data	11 (1)	0 (0)	
Age, No. (%)			
<=40	331 (30)	434 (43)	47 (39)
>40	780 (70)	583 (57)	73 (61)
**Pathology assessment**			
WHO grade, No. (%)			
I	0 (0)	0 (0)	5 (4)
II	249 (22)	291 (29)	34 (28)
III	265 (24)	334 (33)	28 (23)
IV	606 (54)	388 (38)	53 (44)
No data	2 (0)	----	0 (0)
Tumor type, No. (%)			
Astrocytoma, NOS	64 (6)	175 (17)	23 (19)
Astrocytoma, anaplastic	130 (12)	214 (21)	20 (17)
Oligodendroglioma, NOS	112 (10)	112 (11)	3 (3)
Oligodendroglioma, anaplastic	78 (7)	94 (9)	5 (4)
Mixed glioma	131 (12)	30 (3)	16 (13)
Glioblastoma	599 (53)	388 (38)	53 (44)
No data	8 (1)	0 (0)	0 (0)
**Molecular profiles**			
IDH mutation status, No. (%)			
Mutant	91 (8)	531 (55)	----

Wildtype	34 (3)	435 (45)	----
No data	997 (89)	0 (0)	----
1p19q codeletion status, No. (%)			
Codel	----	212 (23)	----
Non-codel	----	728 (77)	----
MGMTp methylation status, No. (%)			
Methylated	----	472 (56)	----
Un-methylated	----	376 (44)	----
**Treatment**			
Radiation therapy, No. (%)			
Treated	759 (68)	202 (21)	----
Untreated	241 (21)	754 (79)	----
No data	122 (11)	0 (0)	
Chemotherapy, No. (%)			
Treated	----	272 (29)	----
Untreated	----	679 (71)	----

Supplementary Table S2. GEO datasets.

	**Status, No. (%)**
**GSE16011**	
Glioma	276 (97)
Control	8 (3)
**GSE7696**	
Non-tumoral	4 (5)
GBM	70 (83)
Recurrent GBM	9 (11)
Re-recurrent GBM	1 (1)
**GSE4290**	
Non-tumor	23 (13)
Astrocytoma, grade 2	7 (4)
Astrocytoma, grade 3	19 (11)

Oligodendroglioma, grade 2	38 (21)
Oligodendroglioma, grade 3	12 (7)
Glioblastoma, grade 4	77 (43)
No data	4 (2)

Supplementary Table S3. IRS results for NID2 and PD-L1 IHC staining using a glioma TMA.

Code	Age at	WHO Grade	Gender	Histological Phenotype	NID2 staini	NID2 positiv	NID2 IRS	PD-L1	PD-L1 positiv	PD-L1 IRS
N0005P0101	Adult	Normal brain tissue	-	-	0	0	negative	0	0	negative
N0007P0104	22	Normal brain tissue	-	-	0	0	negative	0	0	negative
N0008P0100	21	Normal brain tissue	-	-	0	0	negative	0	0	negative
P01A0380	40	WHO I	M	Mixed glioma	0	0	negative	0.5	1	mild
P01A0240	35	WHO I	F	Astrocytoma, NOS	1	0.1	negative	0.5	1	mild
P01A0358	21	WHO I	F	Astrocytoma, NOS	2	0.5	moderate	0.5	0.2	negative
P01A0430	16	WHO I	M	Astrocytoma, NOS	0	0	negative	0.5	0.1	negative
P01A0431	19	WHO I	F	Astrocytoma, NOS	1	0.7	moderate	0.5	1	mild
P01A0315	38	WHO II	M	Oligodendroglioma, NOS	1	0.1	negative	0	0	negative
P01A0324	43	WHO II	F	Oligodendroglioma, NOS	1	0.1	negative	0	0	negative
P01A0336	51	WHO II	M	Oligodendroglioma, NOS	0	0	negative	0.5	1	mild
P01A0298	16	WHO II	M	Astrocytoma, NOS	3	0.4	moderate	1	0.25	mild
P01A0311	14	WHO II	M	Astrocytoma, NOS	2	1	moderate	1	0.1	negative
P01A0248	52	WHO II	F	Astrocytoma, NOS	1	0.2	mild	0	0	negative
P01A0264	35	WHO II	M	Astrocytoma, NOS	2	0.3	moderate	0.5	0.05	negative
P01A0266	16	WHO II	F	Astrocytoma, NOS	2	0.2	moderate	0	0	negative
P01A0273	19	WHO II	M	Astrocytoma, NOS	1	0.1	negative	0.5	0.1	negative
P01A0274	36	WHO II	M	Astrocytoma, NOS	2	0.2	moderate	0	0	negative
P01A0325	37	WHO II	M	Astrocytoma, NOS	3	1	strongly positive	0.5	0.1	negative
P01A0328	26	WHO II	M	Astrocytoma, NOS	2	0.05	mild	0	0	negative
P01A0333	45	WHO II	M	Astrocytoma, NOS	1	0.05	negative	0	0	negative
P01A0356	23	WHO II	F	Astrocytoma, NOS	2	0.3	moderate	1	0.05	negative
P01A0382	36	WHO II	F	Astrocytoma, NOS	2	0.05	mild	0	0	negative

P01A0383	33	WHO II	M	Astrocytoma, NOS	2	1	moderate	0.5	1	mild
P01A0386	46	WHO II	F	Astrocytoma, NOS	2	0.6	moderate	0	0	negative
P01A0449	46	WHO II	M	Astrocytoma, NOS	2	0.6	moderate	0	0	negative
P01A0464	11	WHO II	F	Astrocytoma, NOS	2	0.7	moderate	1.5	0.9	moderate
P01A0471	43	WHO II	M	Astrocytoma, NOS	1	0.1	negative	0.5	0.05	negative
P01A0474	34	WHO II	M	Astrocytoma, NOS	1	0.5	mild	0.5	0.05	negative
P01A0482	40	WHO II	M	Astrocytoma, NOS	2	0.6	moderate	0.5	1	mild
P01A0267	47	WHO II	M	Mixed glioma	0	0	negative	0	0	negative
P01A0275	72	WHO II	F	Mixed glioma	2	0.3	moderate	0.5	1	mild
P01A0341	75	WHO II	M	Mixed glioma	2	0.05	mild	0	0	negative
P01A0357	40	WHO II	F	Mixed glioma	0	0	negative	0	0	negative
P01A0368	37	WHO II	M	Mixed glioma	1	0.1	negative	0.5	0.5	negative
P01A0391	67	WHO II	M	Mixed glioma	1	0.05	negative	0.5	0.1	negative
P01A0400	47	WHO II	F	Mixed glioma	1	0.05	negative	0.5	0.1	negative
P01A0435	36	WHO II	M	Mixed glioma	1	0.05	negative	0	0	negative
P01A0450	44	WHO II	M	Mixed glioma	0	0	negative	0.5	0.1	negative
P01A0459	50	WHO II	F	Mixed glioma	1	0.05	negative	0	0	negative
P01A0462	40	WHO II	F	Mixed glioma	1	0.1	negative	0.5	0.1	negative
P01A0467	40	WHO II	M	Mixed glioma	0	0	negative	0	0	negative
P01A0445	47	WHO II	M	Mixed glioma	1	0.05	negative	1	0.3	mild
P01A0444	48	WHO II	M	Anaplastic oligodendroglioma	3	0.05	mild	0.5	0.2	negative
P01A0254	35	WHO III	F	Astrocytoma, anaplastic	1	0.8	moderate	0.5	0.15	negative
P01A0258	39	WHO III	F	Astrocytoma, anaplastic	2	0.8	moderate	0.5	1	mild
P01A0306	34	WHO III	F	Astrocytoma, anaplastic	2	1	moderate	2	1	moderate
P01A0326	48	WHO III	M	Astrocytoma, anaplastic	3	1	strongly positive	1	0.85	moderate
P01A0329	37	WHO III	M	Astrocytoma, anaplastic	2	0.8	moderate	0.5	1	mild
P01A0436	32	WHO III	F	Astrocytoma, anaplastic	1	0.6	moderate	0.5	1	mild
P01A0239	58	WHO III	F	Anaplastic oligodendroglioma	2	0.05	mild	0.5	0.2	negative
P01A0388	31	WHO III	M	Anaplastic oligodendroglioma	2	1	moderate	1	0.85	moderate
P01A0394	69	WHO III	M	Anaplastic oligodendroglioma	2	0.05	mild	0	0	negative
P01A0465	63	WHO III	F	Anaplastic oligodendroglioma	1	0.9	moderate	1	0.6	moderate
P01A0246	48	WHO III	F	Astrocytoma, anaplastic	2	1	moderate	1.5	0.05	negative

P01A02 52	33	WHO III	F	Astrocytoma, anaplastic	2	0.05	mild	0	0	negative
P01A02 53	64	WHO III	M	Astrocytoma, anaplastic	1	0.05	negative	2	0.7	moderate
P01A02 55	37	WHO III	M	Astrocytoma, anaplastic	2	0.1	mild	0.5	0.1	negative
P01A02 56	51	WHO III	M	Astrocytoma, anaplastic	2	0.1	mild	0.5	0.1	negative
P01A02 61	43	WHO III	F	Astrocytoma, anaplastic	2	1	moderate	1	1	moderate
P01A02 68	44	WHO III	M	Mixed glioma	2	0.4	moderate	0	0	negative
P01A03 09	53	WHO III	M	Astrocytoma, anaplastic	2	0.7	moderate	1	0.6	moderate
P01A03 12	40	WHO III	F	Astrocytoma, anaplastic	3	0.5	moderate	0	0	negative
P01A03 14	36	WHO III	M	Astrocytoma, anaplastic	2	0.8	moderate	0.5	1	mild
P01A03 52	42	WHO III	M	Astrocytoma, anaplastic	2	0.1	mild	0.5	1	mild
P01A03 74	23	WHO III	F	Astrocytoma, anaplastic	3	0.9	strongly positive	1.25	1	moderate
P01A04 41	44	WHO III	F	Astrocytoma, anaplastic	2	0.3	moderate	0.5	0.05	negative
P01A04 48	43	WHO III	M	Astrocytoma, anaplastic	2	0.1	mild	0.5	0.1	negative
P01A04 68	60	WHO III	M	Astrocytoma, anaplastic	2	0.5	moderate	0.5	0.1	negative
P01A04 85	48	WHO IV	M	Glioblastoma	3	0.5	moderate	0.5	0.1	negative
P01A02 49	33	WHO III	F	Mixed glioma	0	0	negative	0	0	negative
P01A02 42	55	WHO IV	M	Glioblastoma	3	1	strongly positive	1	0.5	mild
P01A02 45	46	WHO IV	M	Glioblastoma	3	0.4	moderate	1	0.01	negative
P01A02 57	36	WHO IV	M	Glioblastoma	3	1	strongly positive	0	0	negative
P01A02 60	28	WHO IV	M	Glioblastoma	3	0.5	moderate	1	0.7	moderate
P01A02 69	52	WHO IV	F	Glioblastoma	3	0.2	moderate	1.5	0.01	negative
P01A02 77	56	WHO IV	M	Glioblastoma	3	0.8	strongly positive	1	0.3	mild
P01A03 00	46	WHO IV	M	Glioblastoma	2	0.2	moderate	0.5	1	mild
P01A03 05	67	WHO IV	M	Glioblastoma	3	0.8	strongly positive	1.25	1	moderate
P01A03 07	63	WHO IV	M	Glioblastoma	3	0.2	moderate	2	0.05	mild
P01A03 08	67	WHO IV	F	Glioblastoma	2	0.8	moderate	1	0.01	negative
P01A03 21	41	WHO IV	M	Glioblastoma	2	0.6	moderate	0.5	1	mild
P01A03 23	49	WHO IV	M	Glioblastoma	2	0.1	mild	0.5	0.35	negative
P01A03 30	25	WHO IV	M	Glioblastoma	1	0.1	negative	0.5	0.05	negative
P01A03 31	54	WHO IV	M	Glioblastoma	2	0.1	mild	0.5	0.5	negative
P01A03 35	55	WHO IV	F	Glioblastoma	3	0.7	strongly positive	1	1	moderate
P01A03 40	63	WHO IV	M	Glioblastoma	2	0.7	moderate	1	0.5	mild

P01A03 44	69	WHO IV	M	Glioblastoma	3	0.5	moderate	0.5	0.2	negative
P01A03 53	61	WHO IV	F	Glioblastoma	3	0.3	moderate	1	0.2	mild
P01A03 59	63	WHO IV	M	Glioblastoma	3	0.05	mild	0.5	0.05	negative
P01A03 60	62	WHO IV	M	Glioblastoma	3	0.6	strongly positive	1	0.7	moderate
P01A03 61	50	WHO IV	F	Glioblastoma	2	0.6	moderate	0.5	1	mild
P01A03 62	69	WHO IV	M	Glioblastoma	2	0.8	moderate	0.5	1	mild
P01A03 63	47	WHO IV	M	Glioblastoma	0	0	negative	0.5	0.01	negative
P01A03 65	62	WHO IV	F	Glioblastoma	3	0.8	strongly positive	1	0.5	mild
P01A03 69	42	WHO IV	F	Glioblastoma	3	0.9	strongly positive	1	0.7	moderate
P01A03 71	49	WHO IV	M	Glioblastoma	2	0.7	moderate	0.5	0.5	negative
P01A03 72	46	WHO IV	F	Glioblastoma	3	0.2	moderate	0.5	0.5	negative
P01A03 73	47	WHO IV	M	Glioblastoma	0	0	negative	1	1	moderate
P01A03 75	26	WHO IV	M	Glioblastoma	2	0.8	moderate	1	0.05	negative
P01A03 76	78	WHO IV	M	Glioblastoma	3	0.2	moderate	2	0.8	moderate
P01A03 79	24	WHO IV	F	Glioblastoma	3	0.5	moderate	1	0.35	mild
P01A03 85	33	WHO IV	M	Glioblastoma	2	0.6	moderate	1	0.1	negative
P01A03 96	72	WHO IV	M	Glioblastoma	3	0.2	moderate	0.5	1	mild
P01A03 97	14	WHO IV	M	Glioblastoma	3	0.7	strongly positive	3	1	strongly positive
P01A04 26	65	WHO IV	M	Glioblastoma	3	0.1	mild	0.5	0.05	negative
P01A04 33	47	WHO IV	M	Glioblastoma	3	0.7	strongly positive	0.5	1	mild
P01A04 39	42	WHO IV	M	Glioblastoma	2	0.05	mild	0.5	0.2	negative
P01A04 42	29	WHO IV	M	Glioblastoma	2	0.5	moderate	0.5	0.7	mild
P01A04 51	64	WHO IV	F	Glioblastoma	3	0.5	moderate	1	0.8	moderate
P01A04 53	48	WHO IV	M	Glioblastoma	1	0.4	mild	1.25	0.3	mild
P01A04 55	41	WHO IV	M	Glioblastoma	3	0.2	moderate	0.5	0.5	negative
P01A04 58	68	WHO IV	M	Glioblastoma	3	0.5	moderate	1.25	0.7	moderate
P01A04 60	20	WHO IV	M	Glioblastoma	3	0.2	moderate	1.25	0.8	moderate
P01A04 61	71	WHO IV	M	Glioblastoma	3	0.7	strongly positive	0.5	0.5	negative
P01A04 70	43	WHO IV	F	Glioblastoma	1	0.5	mild	1	0.7	moderate
P01A04 72	55	WHO IV	M	Glioblastoma	3	0.8	strongly positive	1	0.5	mild
P01A04 76	36	WHO IV	M	Glioblastoma	2	0.6	moderate	0.5	1	mild
P01A04 77	64	WHO IV	M	Glioblastoma	0	0	negative	0	0	negative

P01A04 79	42	WHO IV	M	Glioblastoma	3	0.2	moderate	0.5	1	mild
P01A04 80	60	WHO IV	M	Glioblastoma	2	0.2	moderate	1	0.4	mild
P01A04 84	79	WHO IV	M	Glioblastoma	2	0.05	mild	0.5	0.5	negative
P01A04 28	51	WHO IV	M	Glioblastoma	3	0.5	moderate	1.5	0.05	negative
